# Synthesis, Characterization and Anti-Breast Cancer Activity of New 4-Aminoantipyrine-Based Heterocycles

**DOI:** 10.3390/ijms15057539

**Published:** 2014-05-02

**Authors:** Mostafa M. Ghorab, Marwa G. El-Gazzar, Mansour S. Alsaid

**Affiliations:** 1 Department of Pharmacognosy, College of Pharmacy, King Saud University, P.O. Box 2457, Riyadh 11451, Saudi Arabia; E-Mail: msalsaid@ksu.edu.sa; 2 Department of Drug Radiation Research, National Center for Radiation Research and Technology, Nasr City, Cairo 113701, Egypt; E-Mail: marwagalalgazzar@yahoo.com

**Keywords:** pyrazoles, sulfonamides, anti-breast cancer

## Abstract

4-Aminoantipyrine was utilized as key intermediate for the synthesis of pyrazolone derivatives bearing biologically active moieties. The newly synthesized compounds were characterized by IR, ^1^H- and ^13^C-NMR spectral and microanalytical studies. The compounds were screened as anticancer agents against a human tumor breast cancer cell line MCF7, and the results showed that (*Z*)-4-((3-amino-5-imino-1-phenyl-1*H*-pyrazol-4(5*H*)-ylidene)methylamino)-1,5-dimethyl-2-phenyl-1,2-dihydropyrazol-3-one **5**, 3-(4-bromophenyl) -1-(1,5-dimethyl-3-oxo-2-phenyl-2,3-dihydro-1*H*-pyrazol-4-yl)-4-oxo-2-thioxo-1,2,3,4-tetrahydropyrimidine-5-carbonitrile **13**, 1-(1,5-dimethyl-3-oxo-2-phenyl-2,3-dihydro-1-*H*pyrazol- 4-yl)-3-(4-iodophenyl)-4-oxo-2-thioxo-1,2,3,4-tetrahydropyrimidine-5-carbonitrile **14**, 3,3′-(4,4′-sulfonylbis(4,1-phenylene))bis(1-(1,5-dimethyl-3-oxo-2-phenyl-2,3-dihydro-1*H-*pyrazol- 4-yl)-4-oxo-2-thioxo-1,2,3,4-tetrahydropyrimidine-5-carbonitrile) **16**, (*Z*)-1-(1,5-dimethyl-3-oxo-2-phenyl-2,3-dihydro-1*H*-pyrazol-4-yl)-2-hydrazono-4-oxo-3-phenyl-1,2,3,4-tetrahydropyrimidine-5-carbonitrile **17**, (*Z*)-1-(1,5-dimethyl-3-oxo-2-phenyl-2,3-dihydro-1*H*-pyrazol-4-yl)-4-oxo-3-phenyl-2-(2-phenylhydrazono)-1,2,3,4-tetrahydro pyrimidine-5-carbonitrile **18**, and (*Z*)-4-(3-amino-6-hydrazono-7-phenyl-6,7-dihydro pyrazolo[3,4-*d*]pyrimidin-5-yl)-1,5-dimethyl-2-phenyl-1,2-dihydropyrazol-3-one **19** were the most active compounds with *IC*_50_ values ranging from 30.68 to 60.72 μM compared with Doxorubicin as positive control with the *IC*_50_ value 71.8 μM.

## 1. Introduction

Pyrazolone derivatives such as antipyrine, aminopyrine, and dipyrone are well known compounds used mainly as analgesic and antipyretic drugs and their pharmacological molecular mechanism has been widely surveyed [1,2]. One of the best known antipyrine derivatives is 4-aminoantipyrine which is used for the protection against oxidative stress as well as prophylactic of some diseases including cancer, and these are important directions in medical applications [3]. Several derivatives of antipyrine were also biologically evaluated, and analgesic [4], anti-inflammatory [5], antimicrobial [6], and anticancer activity [7–9] have been reported. Antipyrine derivatives are strong inhibitors of cycloxygenase isoenzymes, platelet tromboxane synthesis, and prostanoids synthesis [10], which catalyze the rate-limiting step of prostaglandin synthesis. Pyrazolones are also a well-known elicitor of hypersensitivity [11]. Recently, Al-Haiza *et al.* [12] synthesized a new compound with pyrazolone moiety with antimicrobial and antifungal activities. In the last decade, several pyrazole derivatives proved to have potent anticancer action by the inhibition of the cyclindependent kinases (CDKs) which are members of the large family of protein kinases and are responsible for the eukaryotic cell cycle regulation; they are intensively studied for their cancer implication [13]. Based on the above information and due to our interest in pyrazole as a biologically active pharmacophore [14–18], we synthesized a new series of heterocycles incorporating antipyrine moiety starting from 4-aminoantipyrine to be evaluated for their anticancer activity against human tumor breast cell line (MCF7).

## 2. Results and Discussion

### 2.1. Chemistry

**Scheme 1.**
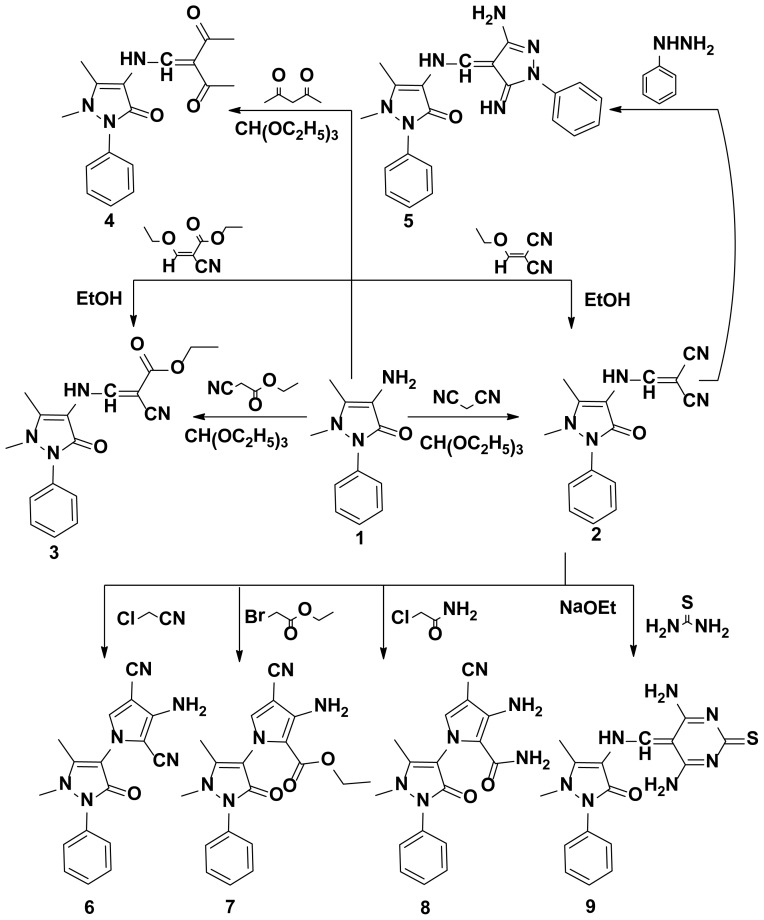
Synthetic pathways for compounds **2**–**9**.

**Scheme 2.**
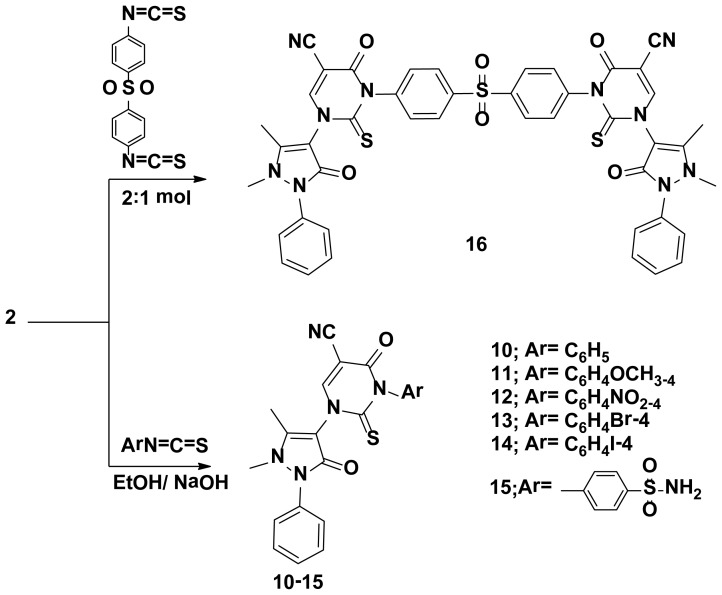
Synthetic pathways for compounds **10**–**16**.

**Scheme 3.**
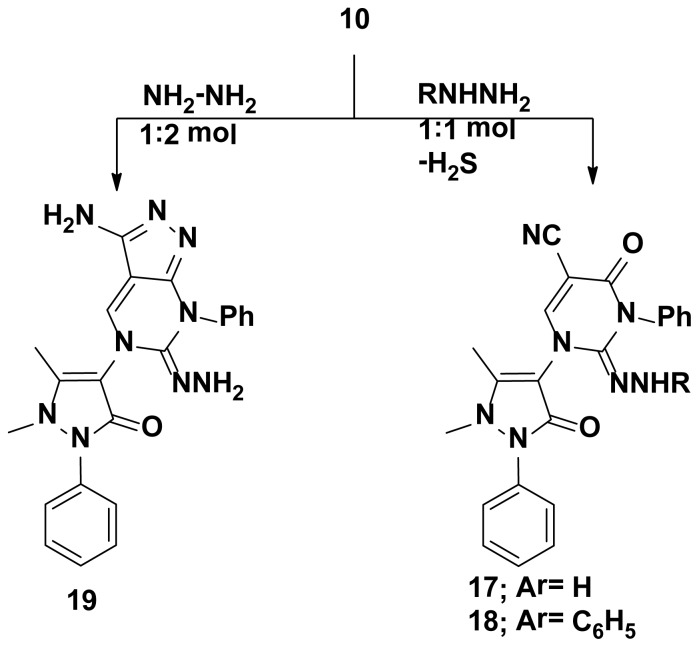
Synthetic pathways for compounds **17**–**19**.

**Scheme 4.**
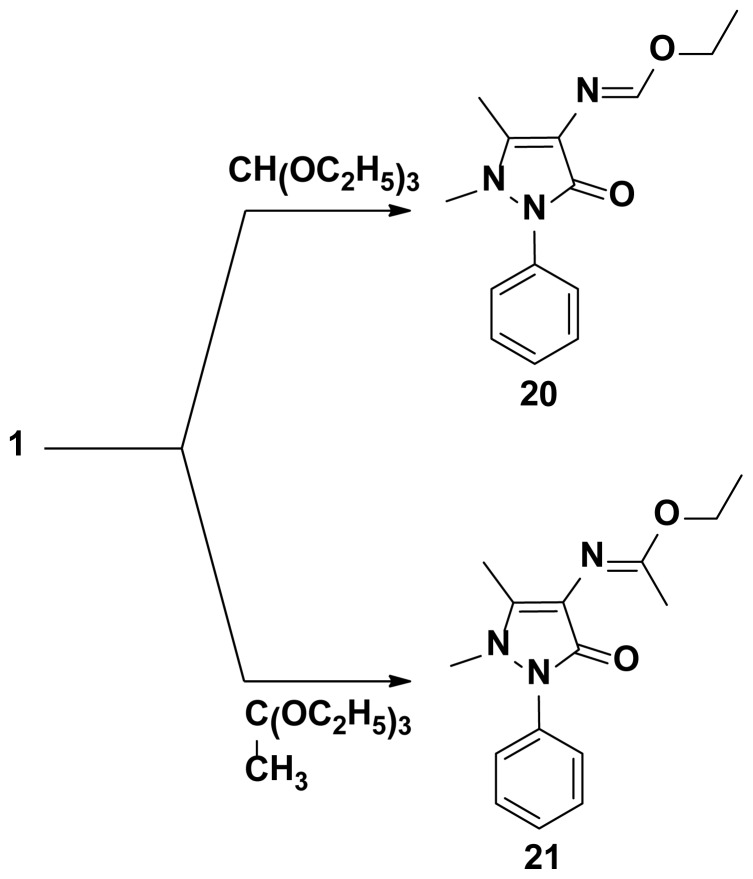
Synthetic pathways for compounds **20**, **21**.

The starting key reagent 4-aminoantipyrine was purchased from Sigma-Aldrich chemical company (St. Louis, MO, USA). In this work, the reactivity of 4-aminoantipyrine with active methylene containing compounds (malononitrile, 2-(ethoxymethylene)malononitrile, ethylcyanoacetate, (ethoxymethylene)ethylcyanoacetate and acetylacetone) was studied and the reaction proceeded in the presence of triethylorthoformate in methanol containing catalytic amounts of acetic acid following the reported reaction condition [19]. The obtained pyrazolone derivatives **2**–**4**, respectively, were identified by elemental and spectral data. Due to the biological importance of pyrazole, pyrrole and pyrimidine rings as anticancer agents [20–22], the pyrazolone derivative **2** was reacted with different nucleophiles in order to obtain biologically active pyrazole **5**, pyrrole **6**–**8** and pyrimidine **9**–**16** derivatives bearing pyrazolone moieties. Thus, interaction of compound **2** with phenyl hydrazine yielded the corresponding pyrazole derivative **5**. On the other hand, interaction of compound **2** with 2-chloroacetonitrile, ethylbromoacetate or 2-chloroacetamide in dioxane containing a catalytic amount of triethylamine yielded the corresponding pyrrole derivatives **6**–**8**, respectively. Reaction of compound **2** with thiourea in refluxing ethanol containing sodium ethoxide gave the corresponding pyrimidine derivative **9** (Scheme 1). The reactivity of compound **2** towards different aryl isothiocyanates/NaOH was studied and the reaction proceeded via an addition reaction onto the isothiocyanato group followed by intramolecular cyclization to produce the pyrimidine ring as in compounds **10**–**15**. Similarly, the bis-compound **16** was obtained expectedly via the reaction of one mole of 1-(4-isothio cyanatophenylsulfonyl)-4-isothiocyanatobenzene with two moles of compound **2** in ethanol containing sodium ethoxide (Scheme 2). The hydrazono pyrimidine derivatives **17**, **18** were synthesized from compound **10** through reaction with either hydrazine hydrate or phenyl hydrazine, respectively, and the reaction proceeded via elimination of H_2_S which was detected by a lead acetate paper. On the other hand, double cyclization took place when one mole of compound **10** was reacted with two moles of hydrazine hydrate to yield the corresponding pyrazolo[3,4-d]pyrimidine **19** (Scheme 3). Finally, treatment of 4-aminoantipyrine **1** with either triethylorthoformate or acetate in acetic anhydride resulted in the formation of compounds **20**, **21**, respectively (Scheme 4). The structures of compounds were proved by microanalytical and spectral data and were consistent with the proposed structures.

### 2.2. In-Vitro Anticancer Screening

Doxorubicin, the positive control used in this study, is an anticancer drug used to treat several cancer diseases including breast cancer. The relationship between surviving fraction and drug concentration was plotted; the response parameters calculated was *IC*_50_ value, which corresponds to the compound concentration causing 50% mortality in net cells (Table 1). The present work describes the synthesis of novel derivatives starting from 4-aminoantipyrine **1** by incorporating biologically active moieties, pyrazole, pyrrole, pyrimidine and pyrazolopyrimidine. From Table 1, we can observe that some of the tested compounds were found to be equipotent or even more potent than Doxorubicin on MCF7 cell line with *IC*_50_ values ranging from 30.68 to 70.65 μM compared to the reference drug (71.8 μM). The most potent compounds in this study were found to be those belonging to the pyrimidine derivatives of antipyrine especially the halogenated ones, where, the iodophenyl **14** (*IC*_50_ = 30.68 μM) and the bromophenyl **13** (*IC*_50_ = 43.41 μM) derivatives showed significant activities, also, the biscompound **16** (*IC*_50_ = 37.22 μM). In addition, pyrazole **5**, pyrimidines **17**, **18**, pyrazolopyrimidine **19** with *IC*_50_ values 60.72, 54.23, 44.99, and 44.49 μM exhibited a higher activity when compared with the Doxorubicin as positive control. The phenyl and 4-nitro phenyl derivatives **10** and **12** (*IC*_50_ = 72.04 and 70.65 μM) are nearly as active as Doxorubicin. On the other hand, reaction of one mole of hydrazine hydrate or phenyl hydrazine with compound **10** yielded the pyrimidine derivatives **17** and **18** with significant activities (*IC*_50_ = 54.23 and 44.99 μM), while, addition of two moles of hydrazine hydrate yielded the pyrazolopyrimidine **19** (*IC*_50_ = 44.49 μM) which is nearly as potent as compounds **17** and **18**. Moreover, the reaction of phenylhydrazine with compound **2** was also successful as it yielded the pyrazole derivative **5** which showed high activity (*IC*_50_ = 60.72 μM). All these compounds are more active than the reference drug. Considering the pyrrole derivatives **6**, **7** and **8**, they were found to exhibit lower activity compared to the reference drug (*IC*_50_ = 128.61, 104.11 and 85.12 μM, respectively). Compounds **2**–**4**, **9**, **11**, **15**, **20** and **21** are the least potent in this study with *IC*_50_ ranging from 109.58 to 173.83 μM.

Generally, incorporation of pyrimidine ring yielded the most potent compounds and these results point to the possible use of pyrimidine derivatives of antipyrine for treatment of breast tumors.

## 3. Experimental Section

### 3.1. General

Reagents were obtained from commercial suppliers and were used without purification. Melting points were determined in open capillary tubes using Thermo system FP800 Mettler FP80 central processor supplied with FP81 MBC cell apparatus (Stuart Scientific, Redhill, UK), and were uncorrected. Elemental analyses (C, H, N) were performed on a Perkin-Elmer 2400 Instrument (Perkin-Elmer, Norwalk, CT, USA). All compounds were within ±0.4% of the theoretical values. Infrared (IR) spectra (KBr disc) were recorded on FT-IR spectrophotometer (Perkin Elmer, Norwalk, CT, USA) at the Research Center, College of Pharmacy, King Saud University, Saudi Arabia.^1^H and ^13^C NMR spectra were recorded on a Ultra Shield Plus 500 MHz (Bruker, Munich, Germany) spectrometer operating at 500 MHz for proton and 125 MHz for carbon, respectively. The chemical shift values are reported in δ (ppm) relative to the residual solvent peak, the coupling constants (*J*) are reported in Hertz (Hz).

### 3.2. Chemistry

#### 3.2.1. 2-((1,5-Dimethyl-3-oxo-2-phenyl-2,3-dihydro-1*H*-pyrazol-4-ylamino)methylene)-malononitrile **2**

A mixture of 4-aminoantipyrine (0.01 mol), malononitrile (0.01 mol), triethylorthoformate (0.01 mol), and acetic acid (1 mL) in methanol (30 mL) was refluxed for 5 h, the reaction mixture was cooled, filtered, the filtered solid was crystallized from ethanol to give **2**. Yield%: 90, m.p. = 254.7 °C, IR, cm^−1^: 3210 (NH), 3055 (CH arom.), 2941, 2818 (CH aliph.), 2200 (CN), 1658 (C=N). ^1^H (DMSO-*d*_6_, ppm): 2.2 [s, 3H, CH_3_], 3.2 [s, 3H, N–CH_3_], 7.3–7.9 [m, 5H, Ar–H], 8.1 [s, 1H, CH], 10.5 [s, 1H, NH, D_2_O-exchangeable]. ^13^C (DMSO-*d*_6_, ppm): 10.3 (CH_3_), 29.1 (N–CH_3_), 50.3, 104.6, 114.1(2), 116.4(2), 117.2, 127.2(2), 129.1, 134.5, 160.1 (C=O), 165.5. Anal. Calcd. for C_15_H_13_N_5_O (279): C, 64.51; H, 4.69; N, 25.07. Found: C, 64.91; H, 4.80; N, 25.34.

#### 3.2.2. (*Z*)-Ethyl-2-cyano-3-(1,5-dimethyl-3-oxo-2-phenyl-2,3-dihydro-1*H*-pyrazol-4-ylamino)acrylate **3**

A mixture of 4-aminoantipyrine (0.01 mol), ethylcyanoacetate (0.01 mol), triethylorthoformate (0.01 mol), and acetic acid (1 mL) in methanol (30 mL) was refluxed for 5 h, the reaction mixture was filtered, the filtered solid was crystallized from ethanol to give **3**. Yield%: 83, m.p. = 154.8 °C, IR, cm^−1^: 3192 (NH), 3065 (CH arom.), 2965, 2841 (CH aliph.), 2214 (CN), 1710, 1658 (2C=O). ^1^H (DMSO-*d*_6_, ppm): 1.2 [t, 3H, CH_3_, *J =* 7.9 Hz], 2.3 [s, 3H, CH_3_], 3.2 [s, 3H, N–CH_3_], 4.2 [q, 2H, CH_2_, *J =* 8.1 Hz], 7.3–8.0 [m, 5H, Ar–H], 8.2 [d, 1H, CH, *J =* 7.12 Hz], 10.0 [d, 1H, NH, D_2_O-exchangeable, *J =* 7.3 Hz]. ^13^C (DMSO-*d*_6_, ppm): 10.3, 14.3, 39.0, 60.2, 72.6, 109.7, 115.7(2), 116.0, 118,1, 129.0(2), 129.1, 134.3, 156.8, 161.0, 166.3. Anal. Calcd. for C_17_H_18_N_4_O_3_ (326): C, 62.57; H, 5.56; N, 17.17. Found: C, 62.90; H, 5.89; N, 17.25.

#### 3.2.3. 3-((1,5-Dimethyl-3-oxo-2-phenyl-2,3-dihydro-1*H*-pyrazol-4-ylamino)methylene)-pentane- 2,4-dione **4**

A mixture of 4-aminoantipyrine (0.01 mol), acetylacetone (0.01 mol), triethylorthoformate (0.01 mol), and acetic acid (1 mL) in methanol (30 mL) was refluxed for 5 h, the reaction mixture was filtered, the filtered solid was crystallized from ethanol to give **4**. Yield%: 90, m.p. = 201.8 °C, IR, cm^−1^: 3209 (NH), 3100 (CH arom.), 2971, 2848 (CH aliph.), 1716, 1692, 1661 (3C=O), 1612 (C=N). ^1^H (DMSO-*d*_6_, ppm): 1.9 [s, 3H, CH_3_], 2.1 [s, 6H, 2COCH_3_], 3.0 [s, 3H, N–CH_3_], 7.3–7.5 [m, 6H, Ar–H + CH], 9.0 [s, 1H, NH, D_2_O-exchangeable]. ^13^C (DMSO-*d*_6_, ppm): 11.1, 22.4(2), 39.1, 104.4, 107.8(2), 108.4, 121.8, 128.8(2), 129.0, 135.0, 151.0, 161.8, 195.1(2). Anal. Calcd. for C_17_H_19_N_3_O_3_ (313): C, 65.16; H, 6.11; N, 13.41. Found: C, 65.87; H, 6.37; N, 13.21.

#### 3.2.4. (*Z*)-4-((3-Amino-5-imino-1-phenyl-1*H*-pyrazol-4(5*H*)-ylidene)methylamino)-1,5-dimethyl-2- phenyl-1,2-dihydropyrazol-3-one **5**

Compound **2** (0.01 mol) was mixed with phenyl hydrazine (0.01 mol) in dioxane (20 mL) and refluxed for 5 h, the reaction mixture was cooled, poured onto ice water. The precipitated solid products were filtered and crystallized from methanol to give compound **5**. Yield%: 76, m.p. = 172.7 °C, IR, cm^−1^: 3386, 3348, 3276 (NH, NH_2_), 3026 (CH arom.), 2981, 2872 (CH aliph.), 1660 (C=O), 1595 (C=N). ^1^H (DMSO-*d*_6_, ppm): 2.2 [s, 3H, CH_3_], 3.2 [s, 3H, N–CH_3_], 7.2–7.9 [m, 10H, Ar–H], 8.3 [s, 1H, CH], 8.7 [s, 1H, NH, D_2_O-exchangeable], 13.2 [s, 1H, NH-imino, D_2_O-exchangeable]. ^13^C (DMSO-d_6_, ppm): 11.5, 39.5, 106.2, 108.0, 112.4(2), 116.1(2), 117.6, 124.1, 127.7(2), 128.9, 129.5(2), 137.9, 141.2, 148.4, 156.5, 163.1, 166.2. Anal. Calcd. for C_21_H_21_N_7_ (387): C, 65.10; H, 5.46; N, 25.31. Found: C, 65.32; H, 5.09; N, 25.55.

#### 3.2.5. 3-Amino-1-(1.5-dimethyl-3-oxo-2-phenyl-2,3-dihydro-1*H*-pyrazol-4-yl)-1*H*-pyrrole-2,4-dicarbonitrile **6**

Compound **2** (0.01 mol) was mixed with 2-chloroacetonitrile (0.01 mol) in dioxane (20 mL) containing 3 drops of triethylamine and refluxed for 5 h, the reaction mixture was cooled, poured onto ice water. The precipitated solid products were filtered and crystallized from methanol to give compound **6**. Yield%: 70, m.p. > 350 °C, IR, cm^−1^: 3344, 3291 (NH_2_), 3100 (CH arom.), 2966, 2881 (CH aliph.), 2200 (CN), 1699 (C=O). ^1^H (DMSO-*d*_6_, ppm): 1.6 [s, 3H, CH_3_], 2.4 [s, 3H, N–CH_3_], 6.5 [s, 2H, NH_2_, D_2_O-exchangeable], 7.0–8.0 [m, 6H, Ar–H + CH-pyrrole]. ^13^C (DMSO-*d*_6_, ppm): 13.9, 39.1, 101.2(2), 107.4, 110.4, 113.9(2), 116.2, 117.6(2), 119.1, 129.3(2), 138.1, 139.4, 161.2. Anal. Calcd. for C_17_H_14_N_6_O (318): C, 64.14; H, 4.43; N, 25.40. Found: C, 64.32; H, 4.67; N, 25.12.

#### 3.2.6. Ethyl-3-amino-4-cyano-1-(1,5-dimethyl-3-oxo-2-phenyl-2,3-dihydro-1*H*-pyrazol-4-yl)-1*H*pyrrole-2-carboxylate **7**

Compound **2** (0.01 mol) was mixed with ethyl bromoacetate (0.01 mol) in dioxane (20 mL) containing 3 drops of triethylamine and refluxed for 5 h, the reaction mixture was cooled, poured onto ice water. The precipitated solid products were filtered and crystallized from methanol to give compound **7**. Yield%: 89, m.p. = 143.4 °C, IR, cm^−1^: 3411, 3396 (NH_2_), 3100 (CH arom.), 2976, 2882 (CH aliph.), 2186 (CN), 1718, 1696 (2C=O). ^1^H (DMSO-*d*_6_, ppm): 1.2 [t, 3H, CH_3_–ester], 2.0 [s, 3H, CH_3_], 2.4 [s, 3H, N–CH_3_], 4.1 [q, 2H, CH_2_–ester], 7.3–8.0 [m, 7H, Ar–H + NH_2_], 8.7 [s, 1H, CH–pyrrole]. ^13^C (DMSO-*d*_6_, ppm): 12.6, 13.4, 39.0, 59.8, 98.7, 112.0, 114.3(2), 115.6, 118.4, 122.7, 126.3, 129.1(2), 138.1, 139.6, 141.2, 160.0, 161.2. Anal. Calcd. for C_19_H_19_N_5_O_3_ (365): C, 62.46; H, 5.24; N, 19.17. Found: C, 62.79; H, 5.57; N, 19.34.

#### 3.2.7. 3-Amino-4-cyano-1-(1,5-dimethyl-3-oxo-2-phenyl-2,3-dihydro-1*H*-pyrazol-4-yl)-1*H*-pyrrole-2- carboxamide **8**

Compound **2** (0.01 mol) was mixed with 2-chloroacetamide (0.01 mol) in dioxane (20 mL) containing 3 drops of triethylamine and refluxed for 5 h, the reaction mixture was cooled, poured onto ice water. The precipitated solid products were filtered and crystallized from methanol to give compound **8**. Yield%: 76, m.p. > 350 °C, IR, cm^−1^: 3404, 3385, 3236 (NH_2_), 3066 (CH arom.), 2961, 2836 (CH aliph.), 2196 (CN), 1700, 1680 (2C=O). ^1^H (DMSO-*d*_6_, ppm): 1.6 [s, 3H, CH_3_], 2.3 [s, 3H, N–CH_3_], 7.2–8.0 [m, 9H, Ar–H + CONH_2_ + NH_2_], 8.5 [s, 1H, CH–pyrrole]. ^13^C (DMSO-*d*_6_, ppm): 11.9, 39.1, 99.2, 111.8, 114.3(2), 115.6, 118.2, 124.4, 129.0(2), 131.8, 135.7, 138.2, 153.2, 162.4, 166.0. Anal. Calcd. for C_17_H_16_N_6_O_3_ (336): C, 60.71; H, 4.79; N, 24.99. Found: C, 60.99; H, 4.49; N, 24.70.

#### 3.2.8. 4-((4,6-Diamino-2-thioxopyrimidin-5(2*H*)-ylidene)methylamino)-1,5-dimethyl-1,2-phenyl-1,2-dihydropyrazol-3-one **9**

A mixture of **2** (0.01 mol) and thiourea (0.01 mol) was refluxed for 5 h in ethanol containing sodium ethoxide (0.01 mol). The reaction mixture was cooled, poured onto ice water, acidified with dil. HCl, the precipitated solid product was filtered and crystallized from methanol to give **9**. Yield%: 90, m.p. = 219.6 °C, IR, cm^−1^: 3410, 3362, 3350 (NH_2_), 3054 (CH arom.), 2922, 2860 (CH aliph.), 1675 (C=O), 1624 (C=N), 1317 (C=S). ^1^H (DMSO-*d*_6_, ppm): 2.2 [s, 3H, CH_3_], 3.1 [s, 3H, N–CH_3_], 7.3 [s, 4H, 2NH_2_, D_2_O-exchangeable], 7.5–7.9 [m, 5H, Ar–H], 9.4 [s, 1H, CH], 13.0 [s, 1H, NH, D_2_O-exchangeable]. ^13^C (DMSO-*d*_6_, ppm): 10.3, 39.3, 84.2, 109.1, 116.4(2), 124.2, 127.2(2), 129.1, 134.4(2), 160.2, 170.8(2), 211.3 (C=S). Anal. Calcd. for C_16_H_17_N_7_OS (355): C, 54.07; H, 4.82; N, 27.59. Found: C, 54.34; H, 4.65; N, 27.25.

### 3.3. General Procedure for Preparation of Compounds **10**–**15**

A mixture of **2** (0.01 mol) and the appropriate aryl isothiocyanate (0.01 mol) was refluxed for 10 h in ethanol containing sodium ethoxide (0.01 mol). The reaction mixture was cooled, poured onto ice water, acidified with dil. HCl, the precipitated solid product was filtered and crystallized from methanol to give **10**–**15**, respectively.

#### 3.3.1. 1-(1,5-Dimethyl-3-oxo-2-phenyl-2,3-dihydro-1*H*-pyrazol-4-yl)-4-oxo-3-phenyl-2-thioxo-1,2,3,4-tetrahydropyrimidine-5-carbonitrile **10**

Yield%: 81, m.p. = 132.9 °C, IR, cm^−1^: 3100 (CH arom.), 2966, 2871 (CH aliph.), 2201 (CN), 1672, 1659 (2C=O), 1292 (C=S). ^1^H (DMSO-*d*_6_, ppm): 2.2 [s, 3H, CH_3_], 3.2 [s, 3H, N–CH_3_], 7.3 [s, 1H, CH–pyrimidine], 7.5–8.1 [m, 10H, Ar–H]. ^13^C (DMSO-*d*_6_, ppm): 14.4, 39.1, 104.6, 109.3, 114.1(2), 116.5, 118.1, 122.0(2), 126.3, 128.6(2), 129.1(2), 129.3, 134.5, 137.8, 139.1, 159.9, 160.5, 187.2 (C=S). Anal. Calcd. for C_22_H_17_N_5_O_2_S (415): C, 63.60; H, 4.12; N, 16.86. Found: C, 63.34; H, 4.32; N, 16.60.

#### 3.3.2. 1-(1,5-Dimethyl-3-oxo-2-phenyl-2,3-dihydro-1*H*-pyrazol-4-yl)-3-(4-methoxyphenyl)-4-oxo-2-thioxo-1,2,3,4-tetrahydropyrimidine-5-carbonitrile **11**

Yield%: 71, m.p. = 90.6 °C, IR, cm^−1^: 3046 (CH arom.), 2991, 2875 (CH aliph.), 2208 (CN), 1684, 1654 (2C=O), 1257 (C=S). ^1^H (DMSO-*d*_6_, ppm): 2.4 [s, 3H, CH_3_], 3.3 [s, 3H, N–CH_3_], 3.7 [s, 3H, OCH_3_], 6.8–7.5 [m, 10H, Ar–H + CH–pyrimidine]. ^13^C (DMSO-*d*_6_, ppm): 13.9, 39.1, 55.1, 95.2, 107.5, 113.5(2), 113.8(3), 123.3, 124.2(2), 124.9, 129.1(2), 130.8, 131.4, 143.6, 156.2, 156.6, 164.7, 188.0. Anal. Calcd. for C_23_H_19_N_5_O_3_S (445): C, 62.01; H, 4.30; N, 15.72. Found: C, 62.34; H, 4.09; N, 15.51.

#### 3.3.3. 1-(1,5-Dimethyl-3-oxo-2-phenyl-2,3-dihydro-1*H*-pyrazol-4-yl)-3-(4-nitrophenyl)-4-oxo-2-thioxo-1,2,3,4-tetrahydropyrimidine-5-carbonitrile **12**

Yield%: 79, m.p. = 190.9 °C, IR, cm^−1^: 3076 (CH arom.), 2991, 2908 (CH aliph.), 2188 (CN), 1700, 1689 (2C=O), 1597, 1380 (NO_2_), 1308 (C=S). ^1^H (DMSO-*d*_6_, ppm): 2.5 [s, 3H, CH_3_], 3.3 [s, 3H, N–CH_3_], 7.8–8.2 [m, 10H, Ar–H + CH–pyrimidine]. ^13^C (DMSO-*d*_6_, ppm): 14.8, 39.3, 89.6, 108.2, 112.3(3), 117.5, 120.9, 124.5(2), 124.9(2), 126.3(2), 129.0, 137.4(2), 142.8, 144.0, 155.6, 162.4, 187.6 (C=S). Anal. Calcd. for C_22_H_16_N_6_O_4_S (460): C, 57.38; H, 3.50; N, 18.25. Found: C, 57.69; H, 3.20; N, 18.56.

#### 3.3.4. 3-(4-Bromophenyl)-1-(1,5-dimethyl-3-oxo-2-phenyl-2,3-dihydro-1*H*-pyrazol-4-yl)-4-oxo-2-thioxo-1,2,3,4-tetrahydropyrimidine-5-carbonitrile **13**

Yield%: 69, m.p. = 76.0 °C, IR, cm^−1^: 3100 (CH arom.), 2986, 2861 (CH aliph.), 2218 (CN), 1684, 1653 (2C=O), 1216 (C=S). ^1^H (DMSO-*d*_6_, ppm): 2.2 [s, 3H, CH_3_], 3.1 [s, 3H, N–CH_3_], 7.0–7.6 [m, 10H, Ar–H + CH–pyrimidine]. ^13^C (DMSO-*d*_6_, ppm): 13.9, 39.5, 92.4, 104.6, 113.8(2), 114.1, 116.6, 119.9, 121.0(2), 127.2(2), 129.0, 132.6, 133.4(2), 137.7, 138.6, 159.9, 160.1, 186.6 (C=S). Anal. Calcd. for C_22_H_16_BrN_5_O_2_S (493): C, 53.45; H, 3.26; N, 14.17. Found: C, 53.70; H, 3.11; N, 14.44.

#### 3.3.5. 1-(1,5-Dimethyl-3-oxo-2-phenyl-2,3-dihydro-1*H*-pyrazol-4-yl)-3-(4-iodophenyl)-4-oxo-2-thioxo-1,2,3,4-tetrahydropyrimidine-5-carbonitrile **14**

Yield%: 90, m.p. = 114.1 °C, IR, cm^−1^: 3092 (CH arom.), 2980, 2861 (CH aliph.), 2187 (CN), 1684, 1654 (2C=O), 1276 (C=S). ^1^H (DMSO-*d*_6_, ppm): 2.5 [s, 3H, CH_3_], 3.1 [s, 3H, N–CH_3_], 7.6–7.9 [m, 10H, Ar–H + CH–pyrimidine]. ^13^C (DMSO-*d*_6_, ppm): 13.9, 39.5, 88.8(2), 107.3, 114.6(2), 116.2, 120.2, 123.6(2), 129.0(3), 130.6, 137.2, 137.9(2), 139.1, 162.7, 166.1, 187.3. Anal. Calcd. for C_22_H_16_N_5_O_2_S (541): C, 48.81; H, 2.98; N, 12.94. Found: C, 48.69; H, 2.69; N, 12.72.

#### 3.3.6. 4-(5-Cyano-3-(1,5-dimethyl-3-oxo-2-phenyl-2,3-dihydro-1*H*-pyrazol-4-yl)-6-oxo-2-thioxo-2,3-tetrahydropyrimidin-1(6*H*)-yl)benzensulfonamide **15**

Yield%: 90, m.p. = 164.8 °C, IR, cm^−1^: 3041 (CH arom.), 2934, 2881 (CH aliph.), 2217 (CN), 1714, 1662 (2C=O), 1296 (C=S). ^1^H (DMSO-*d*_6_, ppm): 2.2 [s, 3H, CH_3_], 3.0 [s, 3H, N–CH_3_], 7.3–8.0 [m, 11H, Ar–H + SO_2_NH_2_], 8.1 [s, 1H, CH–pyrimidine]. ^13^C (DMSO-*d*_6_, ppm): 13.9, 39.4, 96.2, 104.6, 114.2(2), 116.4, 117.2, 121.6(2), 125.1(2), 127.3(2), 129.1, 134.2, 134.5, 139.6, 140.9, 160.2, 160.5, 187.6 (C=S). Anal. Calcd. for C_22_H_18_N_6_O_4_S_2_ (494): C, 53.43; H, 3.67; N, 16.99. Found: C, 53.69; H, 3.90; N, 16.34.

#### 3.3.7. 3,3′-(4,4′-Sulfonylbis(4,1-phenylene))bis(1-(1,5-dimethyl-3-oxo-2-phenyl-2,3-dihydro-1*H-*pyrazol- 4-yl)-4-oxo-2-thioxo-1,2,3,4-tetrahydropyrimidine-5-carbonitrile) **16**

A mixture of **2** (0.01 mol) and 1-(4-isothiocyanatophenylsulfonyl)-4-isothiocyanatobenzene (0.02 mol) was refluxed for 10 h in ethanol containing sodium ethoxide (0.01 mol). The reaction mixture was cooled, poured onto ice water, acidified with dil. HCl, the precipitated solid product was filtered and crystallized from methanol to give **16**. Yield%: 81, m.p. = 140.6 °C, IR, cm^−1^: 3076 (CH arom.), 2981, 2861 (CH aliph.), 2219 (CN), 1661, 1643 (4C=O), 1322 (2C=S). ^1^H (DMSO-*d*_6_, ppm): 2.2 [s, 6H, 2CH_3_], 3.1 [s, 6H, 2N–CH_3_], 7.3–8.0 [m, 18H, Ar–H], 8.6 [s, 2H, 2CH–pyrimidine]. ^13^C (DMSO-*d*_6_, ppm): 13.8(2), 39.0(2), 92.6(2), 108.6(2), 113.5(4), 114.1(2), 118.4(2), 121.7(4), 127.0(4), 128.2(4), 129.1(2), 134.5(2), 136.2(2), 136.7(2), 142.4(2), 159.4(2), 160.2(2), 187.6(2). Anal. Calcd. for C_44_H_32_N_10_O_6_S_3_ (892): C, 59.18; H, 3.61; N, 15.69. Found: C, 59.40; H, 3.80; N, 15.48.

### 3.4. General Procedure for Preparation of Compounds **17** and **18**

Compound **10** (0.01 mol) was mixed with either hydrazine hydrate or phenyl hydrazine (0.01 mol) in dioxane (20 mL) and refluxed for 5 h, the reaction mixture was cooled, poured onto ice water. The precipitated solid products were filtered and crystallized from methanol to give compounds **17** and **18**, respectively.

#### 3.4.1. (*Z*)-1-(1,5-Dimethyl-3-oxo-2-phenyl-2,3-dihydro-1*H*-pyrazol-4-yl)-2-hydrazono-4-oxo-3-phenyl-1,2,3,4-tetrahydropyrimidine-5-carbonitrile **17**

Yield%: 68, m.p. = 78.0 °C, IR, cm^−1^: 3213, 3191 (NH_2_), 3047 (CH arom.), 2961, 2876 (CH aliph.), 2191 (CN). ^1^H (DMSO-*d*_6_, ppm): 2.5 [s, 3H, CH_3_], 3.2 [s, 3H, N–CH_3_], 4.5 [s, 2H, NH_2_, D_2_O-exchangeable], 7.1–7.5 [m, 11H, Ar–H + CH–pyrimidine]. ^13^C (DMSO-*d*_6_, ppm): 13.1, 39.0, 94.6, 106.2, 114.5(2), 116.7, 118.2, 120.6(2), 122.1, 127.6(2), 128.4(2), 129.7, 134.2, 137.7, 138.5, 156.2, 162.8, 168.9. Anal. Calcd. for C_22_H_19_N_7_O_2_ (413): C, 63.91; H, 4.63; N, 23.72. Found: C, 63.70; H, 4.91; N, 23.55.

#### 3.4.2. (*Z*)-1-(1,5-Dimethyl-3-oxo-2-phenyl-2,3-dihydro-1*H*-pyrazol-4-yl)-4-oxo-3-phenyl-2-(2-phenylhydrazono)-1,2,3,4-tetrahydropyrimidine-5-carbonitrile **18**

Yield%: 65, m.p. = 90.6 °C, IR, cm^−1^: 3381 (NH), 3074 (CH arom.), 2966, 2836 (CH aliph.), 2220 (CN), 1700, 1653 (2C=O). ^1^H (DMSO-*d*_6_, ppm): 2.4 [s, 3H, CH_3_], 3.2 [s, 3H, N–CH_3_], 7.0–8.0 [m, 15H, Ar–H], 8.4 [s, 1H, CH–pyrimidine], 9.5 [s, 1H, NH, D_2_O-exchangeable]. ^13^C (DMSO-*d*_6_, ppm): 14.1, 39.6, 95.3, 108.3, 113.8(2), 114.6, 115.8, 116.4(2), 118.9, 119.5(2), 122.2, 128.6(2), 129.0(2), 129.6, 129.8(2), 130.2, 139.2, 141.5, 144.6, 153.5, 155.7, 165.6. Anal. Calcd. for C_28_H_23_N_7_O_2_ (489): C, 68.70; H, 4.74; N, 20.03. Found: C, 68.92; H, 4.99; N, 20.33.

#### 3.4.3. (*Z*)-4-(3-Amino-6-hydrazono-7-phenyl-6,7-dihydropyrazolo[3,4-*d*]pyrimidin-5-yl)-1,5-dimethyl-2-phenyl-1,2-dihydropyrazol-3-one **19**

Compound **10** (0.01 mol) was mixed with hydrazine hydrate (0.02 mol) in dioxane (20 mL) and refluxed for 5 h, the reaction mixture was cooled, poured onto ice water. The precipitated solid products were filtered and crystallized from methanol to give compounds **19**. Yield%: 73, m.p. = 222.6 °C, IR, cm^−1^: 3420, 3362 (NH_2_), 3050 (CH arom.), 2971, 2861 (CH aliph.), 1654 (C=O), 1617 (C=N). ^1^H (DMSO-*d*_6_, ppm): 2.2 [s, 3H, CH_3_], 3.2 [s, 3H, N–CH_3_], 5.4 [s, 2H, N–NH_2_, D_2_O-exchangeable], 6.5 [s, 2H, NH_2_, D_2_O-exchangeable], 7.0–8.1 [m, 11H, Ar–H + CH–pyrimidine]. ^13^C (DMSO-*d*_6_, ppm): 11.4, 39.0, 97.6, 107.3, 112.6(2), 114.3(2), 116.7, 118.6, 123.7, 128.6(2), 129.1, 129.4(2), 134.1, 139.8, 155.7, 162.3, 166.0(2). Anal. Calcd. for C_22_H_21_N_9_O (427): C, 61.81; H, 4.95; N, 29.49. Found: C, 62.03; H, 5.12; N, 29.67.

### 3.5. General Procedure for Preparation of Compounds **20** and **21**

A solution of 4-aminoantipyrine **1** (0.001 mol) in either triethylorthoformate or triethylorthoacetate (0.001 mol) containing three drops of acetic anhydride was refluxed for 8 h, the reaction mixture was cooled and then poured onto cold water, the obtained solid was recrystallized from methanol to give compounds **20** and **21**, respectively.

#### 3.5.1. (*E*)-Ethyl-*N*-1,5-dimethyl-3-oxo-2-phenyl-2,3-dihydro-1*H*-pyrazol-4-yl-formimidate **20**

Yield%: 90, m.p. = 196.9 °C, IR, cm^−1^: 3078 (CH arom.), 2976, 2912 (CH aliph.), 1661 (C=O), 1612 (C=N). ^1^H (DMSO-*d*_6_, ppm): 1.0 [t, 3H, CH_3_], 2.3 [s, 3H, CH_3_], 3.1 [s, 3H, N–CH_3_], 3.5 [q, 2H, CH_2_], 7.2–7.5 [m, 5H, Ar–H], 8.1 [s, 1H, CH]. ^13^C (DMSO-*d*_6_, ppm): 11.3, 18.5, 39.3, 56.0, 106.3, 107.0(2), 120.0, 129.0(2), 135.1, 151.6, 160.1, 164.7. Anal. Calcd. for C_14_H_17_N_3_O_2_ (259): C, 64.85; H, 6.61; N, 16.34. Found: C, 64.66; H, 6.34; N, 16.67.

#### 3.5.2. (*E*)-Ethyl-*N*-1,5-dimethyl-3-oxo-2-phenyl-2,3-dihydro-1*H*-pyrazol-4-yl-acetimidate **21**

Yield%: 88, m.p. = 81.6 °C, IR, cm^−1^: 3086 (CH arom.), 2936, 2817 (CH aliph.), 1658 (C=O), 1598 (C=N). ^1^H (DMSO-*d*_6_, ppm): 1.2 [t, 3H, CH_3_], 1.9 [s, 3H, N=C–CH_3_], 2.1 [s, 3H, CH_3_], 3.3 [s, 3H, N–CH_3_], 4.1 [q, 2H, CH_2_], 7.2–7.5 [m, 5H, Ar–H]. ^13^C (DMSO-*d*_6_, ppm): 10.1 (CH_3_–pyrazole), 14.0 (CH_3_–ethyl), 17.4 (N=C–CH_3_), 39.1 (N–CH_3_), 61.0 (CH_2_–ethyl), 112.6, 118.7(2), 122.8, 128.9(2), 135.4, 146.1, 160.0, 164.0 (C=O). Anal. Calcd. for C_15_H_19_N_3_O_2_ (273): C, 65.91; H, 7.01; N, 15.37. Found: C, 66.13; H, 7.34; N, 15.48.

### 3.6. In-Vitro Anticancer Screening

The human tumor cell line (MCF7) was available from the National Cancer Institute, Cairo, Egypt. The antitumor activity of the newly synthesized compounds against the MCF7 cells was measured using the Sulforhodamine B (SRB) assay by the method of Skehan *et al.* (1990) [23,24]. The cell lines were grown in RPMI 1640 medium containing 10% fetal bovine serum and 2 mM l-glutamine. Cells were plated in 96-multi-well plates (10^4^ cells/well) and were incubated at 37 °C, 5% CO_2_ in a humidified atmosphere for 24 h to allow attachment prior to addition of compounds. The test compounds **3**–**14** were dissolved in DMSO and diluted with saline to the appropriate volume and maintained in RPMI 1640 medium. Different concentrations of the compounds under test were made: 5, 12.5, 25, 30 and 50 μM and were added to the cells. Triplicate wells were prepared for each individual dose. Cells were incubated with the compounds for 48 h at 37 °C, 5% CO_2_. After 48 h, cells were fixed *in situ* by the gentle addition of 50 μL of cold 30% (*w*/*v*) trichloroacetic acid (TCA) (final concentration, 10%) and incubated for 60 min at 4 °C. The supernatant was discarded and the plates were washed five times with tap water and air dried. Sulforhodamine B (SRB) solution (50 μL) at 0.4% (*w*/*v*) in 1% acetic acid was added to each well and plates were incubated for 20 min at room temperature. After staining, unbounded dye was removed by four washes with 1% acetic acid, and attached stain was recovered with Tris-EDTA buffer. Color intensity was measured at wave length 564 nm in an ELISA reader (Gmbh, Viesbaden, Germany). The relation between surviving fraction and drug concentration was plotted to get the survival curve from each compound after the specified time. The concentration required for 50% inhibition of cell viability (*IC*_50_) was calculated and compared with the reference drug doxorubicin and the results are given in Table 1.

## 4. Conclusions

The objective of the present study was to synthesize and investigate the anticancer activity of some novel pyrazole carrying a biologically active sulfonamide moieties. It was found that compounds **5**, **13**, **14**, and **16**–**19** showed promising anticancer activity, higher than that of doxorubicin as reference drug against human breast cancer cell line (MCF7), while compounds **10**, **12** are nearly as active as doxorubicin as positive control. Compound **8** exhibited a moderate activity and compounds **2**–**4**, **6**, **7**, **9**, and **20** showed a weak activity, while compounds **11**, **15** and **21** revealed no activity. Further investigations on different probable mechanisms of action and dose-response studies should be helpful in identifying the specific site(s) of action and optimum doses of the synthesized antipyrine derivatives. These investigations would be crucial in discovering more potent and more selective anti-breast cancer agents.

## Figures and Tables

**Table 1.** *In-vitro* anticancer screening of compounds **2**–**21** against human breast cell line (MCF7).

Compound No.	*IC*_50_ (μg/mL)	*IC*_50_ (μM)
**2**	48.2	173.83
**3**	38.0	116.56
**4**	34.9	111.50
**5**	23.5	60.72
**6**	40.9	128.61
**7**	38.0	104.11
**8**	28.6	85.12
**9**	38.9	109.58
**10**	29.9	72.04
**11**	NA	NA
**12**	32.5	70.65
**13**	21.4	43.41
**14**	16.6	30.68
**15**	NA	NA
**16**	33.2	37.22
**17**	22.4	54.23
**18**	22.0	44.99
**19**	19.0	44.49
**20**	35.8	138.22
**21**	NA	NA

Doxorubicin	39.0	71.8

NA: Compound having *IC*_50_ value > 100 μg/mL.
